# Virtual health assistants: a grand challenge in health communications and behavior change

**DOI:** 10.3389/fdgth.2024.1418695

**Published:** 2024-05-17

**Authors:** Carol Maher, Ben Singh, Allison Wylde, Sebastien Chastin

**Affiliations:** ^1^ Alliance for Research in Exercise Nutrition and Activity (ARENA), University of South Australia, Adelaide, SA, Australia; ^2^ School of Health and Life Sciences, Glasgow Caledonian University, Glasgow, United Kingdom; ^3^ Department of Movement and Sports Sciences, Ghent University, Ghent, Belgium

**Keywords:** digital, chatbot, lifestyle, AI, behavior, ethical considerations, user engagement, health

## 1 Introduction

Modern lifestyles are characterized by convenience and technology. A by-product of this is lifestyle-induced chronic diseases, which are escalating, presenting unprecedented challenges to global health. Worldwide, billions of people are grappling with the repercussions of health-compromising lifestyles—be it poor-quality diets, inadequate physical activity, smoking, or other health-compromising behaviors. Altering entrenched lifestyle patterns is tremendously challenging for individuals and the health system, with most people requiring extensive personalized support to make and sustain lifestyle changes. Such support is prohibitively expensive for many, and public health systems don't have the capacity to deliver the required level of services, making it out of reach for the majority.

eHealth and mHealth lifestyle tools such as AI-powered chatbots have emerged, promising affordable, scalable interventions. However, despite their potential, benefits are often modest, and they frequently fail to sustain user engagement, which is crucial for long-term behavior change. Enter chatbots, which offer the potential of blending the scalability of mHealth technologies with the personalized touch of health coaching. This field, though niche, has shown considerable promise in recent years (1). With the arrival of generative AI tools such as ChatGPT in 2022, new possibilities to meet the complex and personalized requirements of health behavior modification appear vast. This article seeks to explore this new frontier, examining the latest advancements in AI chatbots, how they may revolutionize our approaches to health behavior change, but also acknowledging the challenges that lie ahead, and presenting strategies to help us realize their potential.

## 2 The potential of virtual health assistants

AI-powered chatbots are poised to transform traditional health coaching, offering substantial advantages for fostering healthy lifestyle behaviors through continuous, personalized support. Acting as virtual health assistants (VHAs), chatbots may engage users through real-time conversations, tailored advice, and motivational encouragement. Capable of adapting to an individual's health goals and contexts, VHAs can also offer instant support for someone striving to improve their health routine.

Advanced chatbots and VHA's excel in collecting and analyzing users' data, enabling them to identify patterns and potentially predict lapses in health behaviors. Such capability will enable highly personalized, context-aware interventions, making chatbots a potentially valuable tool for ongoing health coaching. They can leverage data to fine-tune their interactions, ensuring that the guidance remains relevant and engaging over time. Furthermore, they can incorporate elements from various therapeutic approaches, such as motivational interviewing and acceptance and commitment therapy amongst others, potentially enhancing their role as comprehensive virtual health advisors.

Further advantages are that people often find chatbots to be empathetic and non-judgmental (2), which may encourage open disclosure of sensitive information they wouldn't want to share with a human, which may enhance the effectiveness of health interventions. Emerging evidence shows people rate virtual agents as trustworthy counsellors and are willing open up more about stigmatized topics like drug use, depression and sexual symptoms (3, 4). The scalability of chatbots offers the potential to extend health services to a broader audience, overcoming traditional barriers like cost, accessibility, and stigma associated with seeking help.

Clearly, AI chatbots, hold considerable potential to innovate healthcare delivery through the provision of personalized, engaging healthcare. However, these technologies are still in their developmental stages, and realizing this potential fully requires addressing significant challenges.

## 3 Grand challenges

Despite the rapid advances in AI, considerable technical hurdles currently remain. In particular, VHAs can struggle to understand the subtleties of human language and emotion, which is important for effective health coaching (5). Additionally, there's the challenge of ensuring these AI systems provide reliable medical advice across a vast spectrum of individual health scenarios. For example, patients managing multiple chronic diseases, those on medications with specific exercise implications, such as beta blockers, or conditions like poorly controlled diabetes. Furthermore, while sophisticated AI models can retain information throughout extended conversations and across multiple sessions, they predominantly focus on more recent interactions, posing a risk that vital details mentioned earlier may be neglected.

Given that people are likely to disclose personal health information to a VHA, safeguarding the confidentiality and integrity of their data is paramount. Data breaches will have severe consequences, both for the data itself, and undermining public trust, ultimately damaging the viability of AI in healthcare (6). Therefore, VHAs will need to possess inbuilt robust cybersecurity measures that prevent unauthorized data access, while also maintaining acceptable user experience. These issues intersect with regulatory challenges. Virtual health assistants will need to comply with a myriad of laws and regulations, which vary between countries. In particular, many jurisdictions stipulate that electronic health data must not be transmitted overseas, potentially rendering many AI platforms non-compliant. However, these challenges may be overcome, for instance, by developing localized versions of AI platforms that enable data to be processed and stored within the user's country or region. Although local solutions may address some jurisdictional restrictions, problems may increase at scale due to the lack of interoperability between technical processes and governance frameworks as well as moves by some state actors towards internet fragmentation (7).

A range of emerging technical solutions including explainable AI, augmented reality, digital twins, closed systems (8, 9), and synthetic data, offer the potential to address current limitations and increase clinician trust in VHAs. Explainable AI enhances transparency and interpretability, enabling a clearer understanding of how AI decisions are made. Augmented reality can help clinicians interpret complex medical images with greater accuracy, serving as a trusted “second pair of eyes” (9). Digital twins (10)—virtual representations of real-world systems—allow the representations to be modelled to predict the potential behavior of real systems, thereby reducing the probability of failures of security or privacy (10). Closed systems, which use training data and models, address issues of transparency and privacy inherent in open systems. A final approach involves the use of synthetic data to overcome limitations in the availability of real data for training AI systems.

VHAs also raise ethical questions around the replacement of human jobs and the potential loss of personal touch during caregiving. The clinician-patient relationship is integral to the efficacy of care, especially in behavior change, where a patient's motivation is often linked to a sense of accountability to their clinician (11)—an element that might diminish with technology-based programs. While VHAs offer considerable promise for augmenting existing services, or providing services that are currently unavailable, for example, as digital coaches leveraging augmented reality to enhance diagnosis, the economic incentive of replacing human labor with automated systems may be tempting for healthcare organizations aiming to reduce costs. Furthermore, while VHAs have the potential to democratize access to health information and support, like other eHealth programs, they risk inadvertently widening the health disparity gap. This may be particularly an issue for populations with lower digital literacy or those skeptical of digital health.

Finally, for VHAs to be truly effective, they must sustain user engagement and earn users' trust. Whilst latest generation AI platforms offer promise for achieving this, designing AI systems that users feel comfortable and confident in consulting for their health concerns requires a deep understanding of human psychology, behaviors, and needs. There is emerging evidence that users are particularly intolerant of errors made by VHAs or when their questions go unanswered (12), which poses a threat to the long-term engagement with these systems.

## 4 Meeting the challenges

It is easy to imagine that VHAs will develop, as many other digital technologies, organically and at a rapid pace driven by market forces and for-profit private enterprise. It is likely that there will be a growth of an ecosystem that might eventually collapse to a few market leaders with close to a monopoly hold onto the market and service offering, thereby dictating the type of service offered. Left to its own momentum, the development of VHA technology will run ahead of our capacity to regulate them as happened with other digital technologies such as social media and the attention economy, with potential dire consequences. To avoid this dystopian vision, we need to think ahead of some key principles that must guide the development of VHAs.

The first consideration for the deployment of VHAs into public health and healthcare is to ensure they complement rather than substitute human healthcare professionals and services. The focus should be on automating repetitive tasks and maximizing the capacity of VHAs to deal with routine queries and help improve interpretation, thereby freeing up valuable time for healthcare providers while also providing more services. This would allow professionals to focus on more meaningful direct patient care and complex decision-making, at the right time and in areas where the human touch is irreplaceable.

Secondly, VHAs integration into healthcare services. This integration should focus on complementing and enhancing traditional healthcare services rather than being developed as stand-alone solutions. In this respect, VHA should be aimed at weaknesses in current care and prevention systems rather than in reducing systemic issue areas. For example, addressing issues such as the continuous monitoring and maintenance of changes in patient behavior over time, rather than, reducing systemic issues such as waiting lists. Hence, we should think of VHAs as health care modalities that also facilitates easier access to professional help when needed, thus ensuring that VHAs are part of a holistic approach to health and wellbeing rather than merely for user management systems (for example, triage systems).

More importantly, VHAs will have to be developed and deployed in adequacy with the values of health care systems and that at least they “*do no harm”*. Currently, we do not fully understand how AI behaves. Although the science of AI behavior is developing at pace, issues such as a lack of consistency among ethics and governance standards, together with errors such as hallucinations and the amplification of biases and inequalities (13) result in reduced reliability. Improving reliability relies on harnessing the latest AI technological solutions including, for example, the continuous testing of VHA behavior for the emergence of any unintended consequences in their interaction with potentially vulnerable individuals—this is a non-negotiable pre-requisite. The establishment of consistent standards and guidelines for VHAs to ensure that VHAs remain aligned and compliant to the latest medical guidelines and ethical standards is essential (7). As part of this, continuous human monitoring and evaluation are critical for the successful integration of VHAs in lifestyle medicine. Human oversight will also facilitate the adaptation of VHAs to evolving user needs and emerging health trends.

To achieve these goals, the development of VHAs requires a robust interdisciplinary collaboration and co-creation process. Healthcare professionals, AI technology experts, ethicists, legal advisors, and, importantly, the public and end users, need to work together to ensure that VHAs are not only technologically advanced but also ethically sound, compliant with privacy, security and healthcare regulations, and aligned with the real-world needs of users. Healthcare professionals need to provide the necessary clinical expertise, ensuring that the health advice dispensed by VHAs is accurate and safe. AI technology experts need to contribute by refining the training models and algorithms and ensuring the technology is robust and capable of handling complex health data with transparency, securely and efficiently. Ethicists and legal advisors need to help navigate the moral and regulatory landscapes, addressing issues like data privacy, user consent, and potential biases in AI algorithms. End-users need to provide valuable insights into usability and practicality, ensuring that VHAs and their integration into health care and public health are truly user-centric, inclusive and accessible.

User-centered design and co-creation approaches are pivotal in the design and deployment of trusted VHAs. VHAs must be intuitive and responsive to the diverse needs of users. This means considering various user demographics and creating interfaces that are accessible to all, regardless of age, technological literacy, or physical or neurological ability. Personalization is key; each user's unique health journey and preferences should be reflected in the VHA's functionality. This personalization extends beyond mere customization of health recommendations to understanding and adapting to the user's lifestyle, environment, behavioural patterns and interaction with health professionals and health care system. Inclusivity in design also plays a critical role. VHAs should be designed with a wide range of cultural, socio-economic, and health backgrounds in mind. This ensures equitable access to health care advice and interventions, bridging gaps in healthcare disparities.

These fundamental principles should help foster trust and reliability in VHAs, ensuring they are trusted valuable tools rather than replacements for human interaction.

## 5 Conclusion

AI-powered VHAs offer revolutionary potential for lifestyle medicine, providing continuous, personalized support through adaptive conversations to sustain healthy habits. However, realizing this potential requires overcoming technical hurdles around natural language understanding, reliable medical advice, data privacy/security, and regulations. Ethical concerns like replacing human roles, equitable access, and user trust must also be addressed. A thoughtful, interdisciplinary approach guided by key principles is needed: augmenting human providers, integrating with existing care, adhering to healthcare values and standards, continuous monitoring for any unintended impacts, co-creation with users, and fostering trust through intuitive design and leveraging trusted technology. By balancing transformative AI capabilities with human-centric controls and governance frameworks, VHAs can expand access to vital coaching while ensuring quality care. Ultimately, they can enhance healthcare by automating routine tasks so health professionals can focus on meaningful personal interactions where the human touch is indispensable. Carefully developed, AI can improve outcomes while preserving the essential human connection in trusted and effective care.
